# Comparative Study of Relative-Pose Estimations from a Monocular Image Sequence in Computer Vision and Photogrammetry †

**DOI:** 10.3390/s19081905

**Published:** 2019-04-22

**Authors:** Tserennadmid Tumurbaatar, Taejung Kim

**Affiliations:** 1 Department of Information and Computer Sciences, National University of Mongolia, Ulaanbaatar 14200, Mongolia; 2 Department of Geoinformatic Engineering, Inha University, 100 Inharo, Michuhol-Gu, Incheon 22212, Korea; tezid@inha.ac.kr

**Keywords:** object, pose estimation, motion parameters, computer vision, photogrammetry, single camera

## Abstract

Techniques for measuring the position and orientation of an object from corresponding images are based on the principles of epipolar geometry in the computer vision and photogrammetric fields. Contributing to their importance, many different approaches have been developed in computer vision, increasing the automation of the pure photogrammetric processes. The aim of this paper is to evaluate the main differences between photogrammetric and computer vision approaches for the pose estimation of an object from image sequences, and how these have to be considered in the choice of processing technique when using a single camera. The use of a single camera in consumer electronics has enormously increased, even though most 3D user interfaces require additional devices to sense 3D motion for their input. In this regard, using a monocular camera to determine 3D motion is unique. However, we argue that relative pose estimations from monocular image sequences have not been studied thoroughly by comparing both photogrammetry and computer vision methods. To estimate motion parameters characterized by 3D rotation and 3D translations, estimation methods developed in the computer vision and photogrammetric fields are implemented. This paper describes a mathematical motion model for the proposed approaches, by differentiating their geometric properties and estimations of the motion parameters. A precision analysis is conducted to investigate the main characteristics of the methods in both fields. The results of the comparison indicate the differences between the estimations in both fields, in terms of accuracy and the test dataset. We show that homography-based approaches are more accurate than essential-matrix or relative orientation–based approaches under noisy conditions.

## 1. Introduction

The three-dimensional (3D) spatial context is becoming an integral part of 3D user interfaces in everyday life, such as modeling applications, virtual and augmented reality, and gaming systems. The 3D user interfaces are all characterized by a user input that involves 3D position (x, y, z) or orientation (yaw, pitch, roll), and 3D tracking is a key technology to recovering the 3D position and orientation of an object relative to the camera or, equivalently, the 3D position and orientation of the camera relative to the object in physical 3D space. Recovering the position and orientation of an object from images is becoming an important task in the fields of computer vision and photogrammetry. A necessity has arisen for users in both fields to know more about the differences in, and behavior of, the approaches developed by the computer vision and photogrammetry communities. In both fields, mathematical motion models are expressed with an epipolar constraint under perspective geometry. Linear approaches have mainly been developed by the computer vision community, while the photogrammetric community has generally considered non-linear solutions to recover 3D motion parameters.

Especially in computer vision, determining the 3D motion of an object from image sequences starts from image matching, as the establishment of point correspondences extracted from two or more images. The automatic relative orientation of image sequences, with assumptions of calibrated or uncalibrated cameras (unknown intrinsic parameters), has been widely investigated. An essential matrix is defined as the set of linear homogeneous equations found by establishing eight point correspondences. By decomposing the essential matrix, the relative pose parameters of the two perspective views were computed [1,2,3]. Pose estimations based on decomposition of the essential matrix using fewer than eight point correspondences were developed for various applications, such as visual servoing and robot control [4,5,6,7]. Moreover, the pose parameters of the camera relative to a planar object can be estimated by decomposing a homography matrix through point correspondences. Numerical and analytical methods for pose estimation based on homography decomposition were introduced, in detail, in [8]. Other authors introduced non-linear and linear solutions for determining the pose parameters based on the decomposition of a homography matrix in augmented reality and robot control applications [9,10,11,12,13,14].

In photogrammetry, the determination of 3D pose from a set of 3D points was originally known as resectioning. To determine the position and orientation (extrinsic parameters) of the right image, relative to the left image, from a sufficient set of tie-points is well known as a relative orientation process. In this photogrammetric task, the intrinsic parameters are assumed to be known, instead the analysis of the images, to discover the corresponding points between the images, but no ground truth is assumed. Mathematically, relative orientation parameters, as pose parameters between frames in a sequence, can be determined by collinearity or coplanarity equations [15,16,17,18,19].

Various solutions for pose estimation from the point correspondences have been published, introducing their accuracy to image noise and their computation speed against state-of-the-art methods for both simulated and real image data. The non-linear and the linear approaches to solve the perspective-n-point (PnP) problem for determining the position and orientation of the camera based on the 3D reference points and their 2D projections have been developed for various applications, and quantitative comparisons of their results with the state-of-the-art approaches were carried out. The authors in [20] developed an algorithm to solve the PnP problem using the 3D collinearity model. The experimental results on simulated and real data were compared with the efficient PnP (EPnP) algorithm [21], the orthogonal iterative (OI) approach [22], and a non-linear solution to relative position estimation [23]. An algebraic method was developed, in [24], to solve the perspective-three-point (P3P) problem of computing the rotation and position of a camera. The experimental results demonstrated that the accuracy and precision of the method was comparable to the existing state-of-the-art methods, such as the first complete analytical solution to the P3P problem [25], the perspective similar triangle (PST) method [26], and the closed-form solution to the P3P problem [27]. The authors in [28] proposed a method for the PnP problem, for determining the position and orientation of a calibrated camera from known reference points. The authors investigated the performance of the proposed method and compared the accuracy with the leading PnP methods, such as the non-linear least-squares solution [29], the efficient PnP (EPnP) algorithm [21], the robust non-iterative method (RPnP) [26], the direct least-squares (DLS) method [30], and the optimal solution (OPnP) [31]. A novel set of linear solutions to the pose estimation problem from an image of *n* points and *n* lines was presented in [32]. Their results were compared to other recent linear algorithms, such as the non-linear least-squares solutions [29,33], the direct recovery and decomposition of the full projection matrix [34], and the *n* point linear algorithms [35,36]. Combining two fields, a evaluation of epipolar resampling methods developed for image sequences with an intension of stereo applications was reported in [37]. A theoretical comparison of the 3D metric reconstruction in the cultural heritage field and its accuracy assessment with the results from commercial software were discussed in [38]. The relationship between photogrammetry and computer vision was examined in [39]. However, the evaluations, comparisons, and the differences in the theoretical and practical perspectives of pose estimation methods based on the epipolar constraint developed in each field, using a common dataset, are missing. On the other hand, even if the processing steps for the two techniques seem to be different approaches, some computer vision techniques were implemented for increasing the workflow automation of the photogrammetric techniques. Moreover, an additional investigation of the automatic techniques that regulate the processing steps is needed.

The aim of this paper is to investigate the approaches to determining the 3D motion of a moving object in real-time image sequences taken by a monocular camera. First, we determined a novel automatic technique to perform motion estimations with a single camera. Common processing steps were implemented in the estimations. We try to use a web camera instead of other specialized devices, such as accelerometers, gyroscopes, and global positioning systems (GPS), to sense 3D motion, as the web camera is cheap and widely available. Furthermore, we believe a single camera is more suitable for real-time computation, because we can maintain one processing chain. Second, we analyzed and compared the linear approaches with the non-linear approaches. We used four estimation methods for recovering the 3D motion parameters, based on tracked point correspondences. Two of them were developed with linear solutions in computer vision, and two of them were developed with non-linear solutions in photogrammetry. We argue that most previous investigations for pose estimations have not thoroughly compared the methods developed in both fields. It is, at present, difficult to define the border between the two methodologies. Moreover, a number of applications have recently been developed by linking the techniques developed in both fields. It is, thus, important to show the differences in the methods and to confirm which one is the most suitable technique to be used, based on the needs of the given application. We aim to identify the differences and better understand how those differences may influence outcomes. First, we review general principles and mathematical formulations for motion models in linear and non-linear solutions. Next, we explain the implementation steps of pose estimations, regarding image sequences from a single camera. Third, we point out the practical differences at the experimental level, with the test datasets, and analyze the results. This paper is organized as follows. Mathematical models of the proposed methods in computer vision and photogrammetry are described in Section 2. Processing techniques to implement pose estimations in both fields, with test datasets, are presented in Section 3. The comparison results are discussed in Section 4. The conclusions are presented in Section 5.

## 2. General Motion Model

We reviewed the basic geometry of the proposed motion models to determine the relative pose of a moving object in image sequences, once the correspondence points have been established. We assumed that a stationary camera has taken an image sequence of the moving object through its field of view. The camera coordinate system is fixed with its origin, *O*, at the optical center. The *z* axis is pointing in the direction of the view, coinciding with the optical axis. The image plane is located at a distance equal to the focal length, which is considered to be unity.

**Figure 1:**
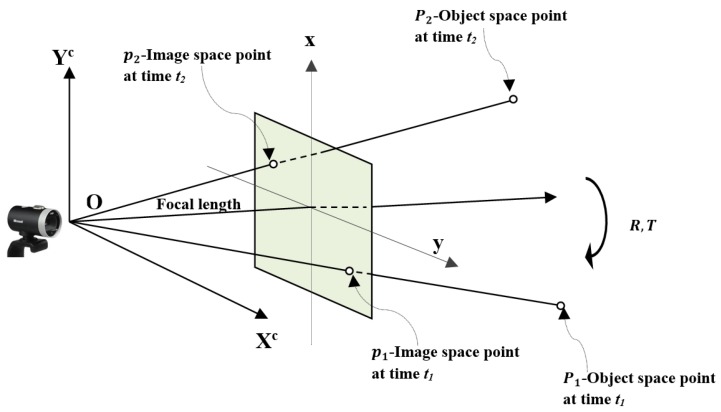
Geometry of the imaging system.

Consider point $P_{1}$ on a rigid object at time $t_{1}$ moving to point $P_{2}$ on a rigid object at time $t_{2}$, with respect to the camera coordinate system (as shown in Figure 1). Point $P_{1}$ is projected at point $p_{1}$ on the image plane under perspective projection. Similarly, point $P_{2}$ is projected at point $p_{2}$ on the image plane. The object-space coordinates of point $P_{1}$ are $X_{1}\in R^{3}$, and the image-space coordinates are defined as $x_{1}\in R^{3}$. The object-space coordinates of point $P_{2}$ are $X_{2}\in R^{3}$, and the image-space coordinates are defined as $x_{2}\in R^{3}$.

To summarize, our problem is as follows: 

*Given two image views with correspondences $\left(p_{1},p_{2}\right)$, find 3D rotations and 3D translations.*

We have that $P_{1}$ and $P_{2}$ are related by the rotation matrix *R* and translational vector *T*, due to the rigidity constraint of the object motion:

(1) $$X_{2}=RX_{1}+T.$$

It is obvious, from the geometry of Figure 1, that $Rx_{1}$, $x_{2}$, and *T* are coplanar, which can be written in matrix form as

(2) $$x_{2}^{T}\hat{T}Rx_{1}=0$$

This is called coplanarity, or an epipolar constraint, written in a triple product of vectors.

### 2.1. Recovering Motion Parameters from an Essential Matrix

We can reformulate Equation (2) as follows:

(3) $$x_{2}^{T}Ex_{1}=0,$$

where

$$E=\hat{T}R\in R^{3x3}\;\mathrm{and}\;\hat{T}=\left(\begin{array}{ccc} 0 & -B_{z} & B_{y}\\ B_{z} & 0 & -B_{x}\\ B_{y} & B_{x} & 0 \end{array}\right).$$

Equation (3) is linear and homogeneous in the nine unknowns. Given *N* point correspondences, we can write Equation (3) in the form:

(4) $$A_{N}{\left[e_{1},e_{2},e_{3},e_{4},e_{5},e_{6},e_{7},e_{8},e_{9}\right]}^{T}=0.$$

The relative pose between two views can be found from matrix Equation (3), encoded by a well-known essential matrix. Due to the theorem in [40], *E* has singular value decomposition (SVD), defined as:

(5) $$E=U\Sigma V^{T},$$

where *U*, *V*, and Σ are chosen such that det(U)>0, det(V)>0, and $\Sigma =\left\{1,1,0\right\}$. Furthermore, the following formulas give the two distinct solutions for the rotation and translation vectors from, the essential matrix.

(6) $$R=U\left[\begin{array}{ccc} 0 & \mp 1 & 0\\ \pm 1 & 0 & 0\\ 0 & 0 & 1 \end{array}\right]V^{T},\;\hat{T}=U\left[\begin{array}{ccc} 0 & \mp 1 & 0\\ \pm 1 & 0 & 0\\ 0 & 0 & 1 \end{array}\right]U^{T}.$$

One of the four possible solutions corresponds to the true solution for Equation (6). The correct solution can be chosen by enforcing a constraint called the cheirality test [41]. The cheirality test basically means that the triangulated scene points should have positive depth, and scene points should be in front of the camera.

### 2.2. Recovering Motion Parameters from a Homography Matrix

**Figure 2:**
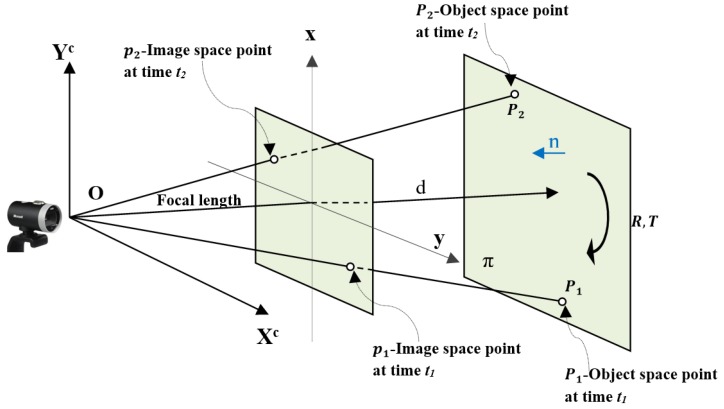
Geometry of a planar object in an image sequence.

Consider the points $P_{i},i=1,2$ to be on a 2D plane, π, in 3D space. The plane π has unit normal vector $n=\left[n_{1},n_{2},n_{3}\right]$, and d(d>0) denotes the distance from plane π to the optical center of the camera. Suppose the optical center of the camera never passes through plane π, as shown in Figure 2.

Then, we formulate the following equation, normalizing the translational vector *T* by the plane depth *d* from Equation (1):

(7) $$\begin{array}{c} \begin{array}{c} X_{2}=RX_{1}+T=\left(R+\frac{1}{d}Tn^{T}\right)X_{1}=HX_{1}\\ H=\left(R+\frac{1}{d}Tn^{T}\right)\in R^{3x3} \end{array} \end{array}.$$

We call the matrix H a planar homography matrix, as it depends on motion parameters $\left\{R,T\right\}$ and structure parameters $\left\{n,d\right\}$. We have a homography mapping induced by the plane π, since it denotes a linear transformation from $X_{1}\in R^{3}$ to $X_{2}\in R^{3}$, due to the scale ambiguity in the term $\frac{1}{d}T$ in Equation (7):

(8) $$x_{2}∼Hx_{1}.$$

Equation (8) is linear and homogeneous in the nine unknowns. Given *N* point correspondences, we can write Equation (8) in the form:

(9) $$B_{N}{\left[h_{1},h_{2},h_{3},h_{4},h_{5},h_{6},h_{7},h_{8},h_{9}\right]}^{T}=0.$$

After we have recovered the *H* matrix, using at least four point correspondences, we can decompose the matrix into its motion and structure parameters by SVD ([8,42,43,44]):

(10) $$H=U\Sigma V^{T},$$

where *U* and *V* are orthogonal matrices, and Σ is a diagonal matrix which contains a singular value for *H*. Then, we also obtain four solutions: Two completely different solutions, and their opposites for decomposing the *H* matrix. In order to reduce the number of physically possible solutions, we impose a positive depth constraint, having $n^{T}e>0,e=\left[0,0,1\right]$, as the camera can see only points in front of it.

### 2.3. Recovering Motion Parameters from Relative Orientation

In photogrammetry, determining the relative position and orientation of the first view of the frame, with respect to the next view of the frame in a sequence, is well-known as a relative orientation process. In general, the geometric relationship between a ground point *P* and its corresponding image points, $p_{1}$ and $p_{2}$, at two time instants, is formulated by the coplanarity constraint from Equation (2). As shown in Equation (2), the essential matrix determined in computer vision is mathematically identical to a coplanarity condition equation in photogrammetry, which has been well-confirmed [19,37].

By equivalently reformulating the non-linear equations of the coplanarity condition in [17] into Equation (2), we have the following:

(11) $$\begin{array}{c} \left[\begin{array}{c} D_{1}\\ E_{1}\\ F_{1} \end{array}\right]=\left[\begin{array}{c} ix_{1}\\ jx_{1}\\ kx_{1} \end{array}\right],\;\left[\begin{array}{c} D_{2}\\ E_{2}\\ F_{2} \end{array}\right]=\left[\begin{array}{c} ix_{2}\\ jx_{2}\\ kx_{2} \end{array}\right],\\ b=\left[\begin{array}{ccc} B_{x} & B_{y} & B_{z} \end{array}\right],\;R=\left[\begin{array}{ccc} i_{x} & j_{x} & k_{x}\\ i_{y} & j_{y} & k_{y}\\ i_{z} & j_{z} & k_{z} \end{array}\right], \end{array}$$

where

$\begin{array}{c} i_{x}=\cos\alpha *\cos\kappa ;\;j_{x}=-\cos\phi *\cos\kappa ;\\ k_{x}=\sin\phi ;\\ i_{y}=\sin\omega *\sin\phi *\cos\kappa +\cos\omega *\sin\kappa ;\;j_{y}=-\sin\omega *\sin\phi *\sin\kappa +\cos\omega *\cos\kappa ;\\ k_{y}=-\sin\omega *\cos\phi ;\\ i_{z}=-\cos\omega *\sin\phi *\cos\kappa +\sin\omega *\sin\kappa ;\;j_{z}=\cos\omega *\sin\phi *\sin\kappa +\sin\omega *\cos\kappa ;\;\mathrm{and}\\ k_{z}=\cos\omega *\cos\phi . \end{array}$

Then, the triple-scalar product of the three vectors is as follows:

(12) $$B_{x}\cdot \left(E_{1}F_{2}-E_{2}F_{1}\right)+B_{y}\cdot \left(F_{1}D_{2}-F_{2}D_{1}\right)+B_{z}\cdot \left(D_{1}E_{2}-D_{2}E_{1}\right)=0.$$

**Figure 3:**
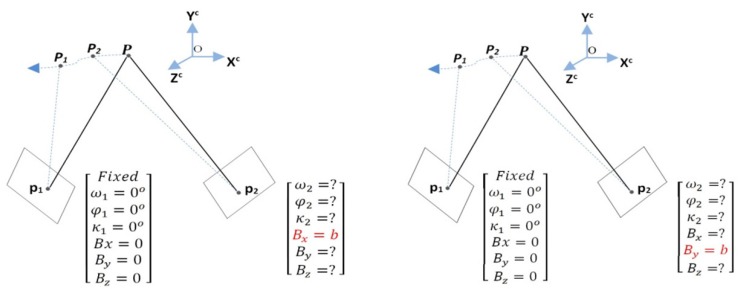
Parameter setup configurations of relative orientation-based estimations.

From this non-linear equation, the unknowns can be obtained by Taylor’s linearization in a least-squares solution. The three parameters of the rotation matrix *R*, and two components of the base vector, are estimated. In Figure 3, ω, ϕ, and κ are rotations about the *x*, *y*, and *z* axes, respectively; and $B_{x}$, $B_{y}$, and $B_{z}$ are translations about the *x*, *y*, and *z* axes, respectively. The iterative method for solving a non-linear equation requires an initial estimate for each unknown parameter for convergence to the correct answer. We set the position and orientation angles of the first-view by making all six variables equal to zero ($\omega_{1}=\phi_{1}=\kappa_{1}=0^{\circ },\;B_{x}=B_{y}=0$), because we considered no movement of the object in the first-view. The second-view orientation is set as $\omega_{2}=\phi_{2}=\kappa_{2}=0^{\circ }$, by assuming a constant fixed value for $B_{x}$ or $B_{y}$ based on simple parallax differences between the two views, as is illustrated in Figure 3.

### 2.4. Recovering Motion Parameters from Homography-Based Relative Orientation

By reformulating matrix Equation (8), we can obtain the motion and structure parameters from the following non-linear equation, as mapping from the first to the next image is given by homography:

(13) $$x_{2}≅\left(R+\frac{1}{d}Tn^{T}\right)x_{1}.$$

The seven unknown parameters of this equation can be described as five parameters (ω, ϕ, κ, $B_{x}$ or $B_{y}$, and $B_{z}$) for the relative orientation, and two parameters $\left(n_{1},n_{2}\right)$ for the plane normal in object-space. Similarly, this equation can be solved by Taylor’s linearization in a least-squares solution, with an a priori fixed value for $B_{x}$ or $B_{y}$, as illustrated in Figure 3. We set the initial value for the variable $n_{3}$ as 1.

### 2.5. Summary of Approaches

We have reviewed estimations for determining the motion of a rigid body from 2D to 2D point correspondences. If the rank of $A_{N}$ in Equation (4) is 8, *E* can be determined uniquely, within a scale factor, and with an eight point correspondence. Once *E* is determined, *R* and *T* can be determined uniquely. If the rank of $A_{N}$ is less than 8, then either we have a pure rotation case or the surface assumption of eight points is violated. However, the surface assumption (position) of the given eight points is very important. It can be easily shown that, if these points form a degenerate configuration, the estimation will fail [44]. This approach is sensitive to noise. Pure rotation of the object in the images generates numerical ill-conditioning, so enough translation in the motion of the object is needed in the images for the estimation (given in Section 2.1) to operate correctly. With a four point correspondence, the rank of $B_{N}$ in Equation (9) is 8 when the 3D points lie on a plane [45]. In this case, a pure rotation can be handled by the planar approach. Then, we have a unique solution for *H*, within a scale factor. Once *H* is determined, *R* and *T* can be determined uniquely. If the rank of $B_{N}$ is more than 8, it is considered that the 3D points do not lie on a plane. As both *E* and *H* give four possible solutions to (R,T), the depths of the 3D points being observed by the camera are all positive. Therefore, one of the four solutions will be chosen, based on the positive depth constraint. To find a least-squares solution of the non-linear equations in (12) and (13), using the iterative method is not computationally expensive; however, we need a good initial value for its convergence to the correct solution. We set the initial values for $B_{x}$ or $B_{y}$ differently, depending on the experimental settings. However, we expect that the solution for non-linear approaches is generally unique with six or more point correspondences for the five motion parameters.

## 3. Implementation Steps for Estimations and Datasets

### 3.1. Methodology

**Figure 4:**
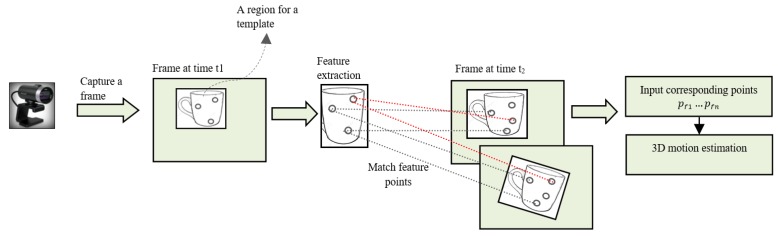
Process flow for implementing the estimations.

In this section, we explain the processing steps for implementing the four estimations and how these have to be considered with a single camera. Figure 4 provides the process flow for implementing the estimations. We estimate the position and orientation of the moving object from two views, such as between a template region and the next consecutive frames, using point correspondences.

First, an initial frame is captured at time $t_{1}$, and a template region is extracted from it. Then, feature points for the extracted template region are computed by using a scale-invariant feature transform (SIFT) feature extractor. The template region is a rectangle, in which the extracted points are circles in a frame at time $t_{1}$, as shown in Figure 4. SIFT features are robust to perspective changes. This advantage is preserved in the matching process, as well. Here, we assume that *n* feature points are extracted to the template region and tracked through each of the next *f* frames. In other words, template extraction could be done automatically by analyzing moving parts of the object between frames through these feature points.

Secondly, after capturing the next frame of the moving object at time $t_{2}$, feature points of the next frame are extracted. Feature points in the next frame are matched with feature points in the template, as described in Figure 4. The best matches for corresponding points between the two views are found by a Brute-Force matcher. Outliers among the matched points are eliminated by a random sample consensus (RANSAC) method [46] before estimation of the motion parameters. We used different RANSAC methods for each of the four proposed approaches. Homography-based RANSAC was applied before estimation of the motion parameters from homography-based methods in both fields. Essential matrix-based RANSAC was applied before estimation of the motion parameters from an essential matrix. Relative orientation-based RANSAC was applied before estimation of the motion parameters from the relative-orientation method.

Third, when all processing steps are accumulated, as explained above, the proposed estimations (as defined in Section 2) are used to estimate the motion parameters.

### 3.2. Test Datasets

We implemented the estimations in both fields with Visual C++, Open Source Computer Vision Library (OpenCV) 2.4.9, and Open Graphics Library (OpenGL) on a PC with an Intel Core i5 CPU at 3.0 GHz with 4096 MB of RAM and a Microsoft LifeCam. The camera’s intrinsic parameters, including focal length, principle point, and lens distortion coefficients, were determined with the GML Camera Calibration Toolbox [47].

We experimentally examined and compared the performance of estimations with a real dataset (created from real scenes) and a simulated dataset (created from an OpenGL library). Video sequences for the moving object were captured at a resolution of 640×360 pixels.

For creation of the real dataset, we captured video sequences while changing object positions along each axis and rotating the object around each axis in front of a static camera. Note that the object position in the initial frame was arbitrarily fixed before changing it. Translation of the object along the *x* and *y* axes varied by up to 200 mm. Translation of the object along the *z* axis varied between 300–800 mm from the camera. Rotations of the object around the *x* and *y* axes varied by up to 20 degrees, and rotation of the object around the *z* axis varied by up to 90 degrees. We used thousands of image sequences, comprised of different textured 3D objects and planar objects, to check the accuracy of estimations of the motion parameters. Each object template was composed of approximately one hundred frames. Object templates used in the experiments and their feature descriptions are listed in Table 1.

For creation of the simulated dataset, we used different 3D objects, such as polygons, pyramids, and cubes with different edge lengths, by creating them in Euclidean space. We manually measured up to 25 feature points on the 3D objects. The simulated sequences for moving 3D objects were created through perspective projection by keeping the object in the field of view throughout the sequences. Descriptions of the simulated dataset are summarized in Table 1. Translations of the 3D objects along the *x*, *y*, and *z* axes varied by up to 15 units. Rotations of the 3D objects around the *x* and *y* axes varied by up to 30 degrees. Rotation around the *z* axis varied by up to 90 degrees. We used thousands of simulated sequences for the experiments. Each 3D object template was composed of approximately one hundred frames.

## 4. Performance of 3D Motion Estimation

### 4.1. Performance Analysis for Estimations with a Real Dataset

After estimating the motion parameters, we analyzed the accuracy of the proposed four estimations in Section 2. We renamed the estimation methods to simplify the notation in the experimental results. Decomposition of the essential matrix is notated as (CV_E). Decomposition of the homography matrix is notated as (CV_H). Relative orientation is notated as (PM_RO). Homography-based relative orientation is notated as (PM_H).

First, we estimated the motion parameters for the real dataset in two different cases. We checked the accuracy of the estimated rotation parameters by comparing them with true (known) rotation parameters. For a true reference value, we manually measured the corresponding points between the template and the next consecutive frames of the object. Using the measured corresponding points, the precise 3D motion was estimated for each frame. In the first case, the object in the image sequences was rotated along only one of the *x*, *y*, or *z* axes separately, and translated along one of the axes. In the second case, we estimated the motion parameters of an object from image sequences rotated by a combination of rotations around the *x*, *y*, and *z* axes, and translated along an *x*, *y*, or *z* axis differently. For example, the object was rotated around the *y* axis by $5^{\circ }$, and simultaneously rotated around the z axis by up to $25^{\circ }$.

**Figure 5:**
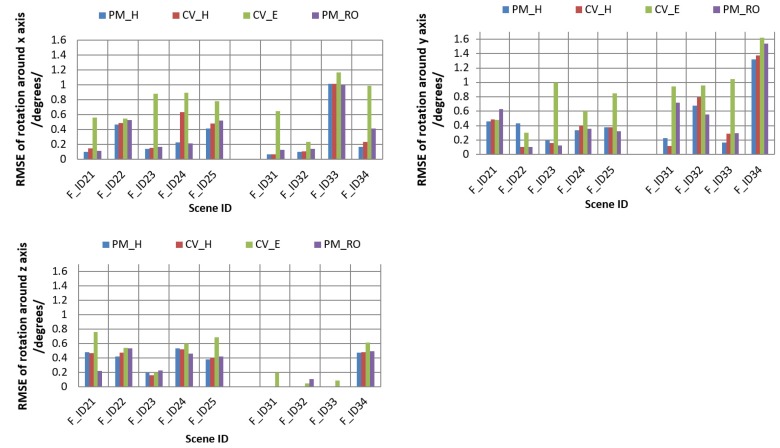
Comparisons of error analyses for rotations around the *x*, *y*, and *z* axes for real scenes.

For both cases, we analyzed the mean, maximum (Max), and minimum (Min) errors for the four estimations, as summarized in Table 2. We also checked the accuracy of the estimated rotation parameters around each of the three axes by comparing them with true (known) rotation parameters and analyzing the root mean square error (RMSE). The RMSEs for rotation around the *x*, *y*, and *z* axes are shown in Table 3, Table 4 and Table 5, respectively; which are summarized for each dataset used in the experiments. A graphical representation of the comparisons is shown in Figure 5.

As we can see, from Table 2, Table 3, Table 4 and Table 5, the RMSEs were small, and the rotation results were accurate for each of the four approaches in both of the test cases. In particular, the homography-based methods PM_H and CV_H produced more accurate results for image sequences of the moving planar object, since the planar pattern is dominant in the test datasets F_ID31 to F_ID34. On the other hand, PM_H and CV_H produced more negligible and comparable errors, among the four methods with noisy correspondences for the datasets of both 3D and planar objects. Among them, the motion parameters from PM_H estimations are especially accurate. We observe that the estimation errors for the motion parameters slightly increased in the dataset for cases F_ID33 in Table 3 and F_ID34 in Table 4, due to noisy feature correspondences. For these datasets, the object was translated close to the camera along the z axis and was rotated around the x or y axis to a large degree. Object rotation around the x or y axis at large degrees creates the side-effect of scaling. In this case, matched feature correspondences are noisy and unstable in their matched positions. Generally, we can see that the CV_E estimation method was sensitive to noisy measurements in feature correspondences, as is seen in most of the results in Table 2, Table 3, Table 4 and Table 5. Another interesting result is that the motion parameters estimated by PM_RO were the most accurate, compared to the other three approaches for the 3D object datasets F_ID21 to F_ID25.

#### Stability Analysis for Estimations with Varying Noises

**Figure 6:**
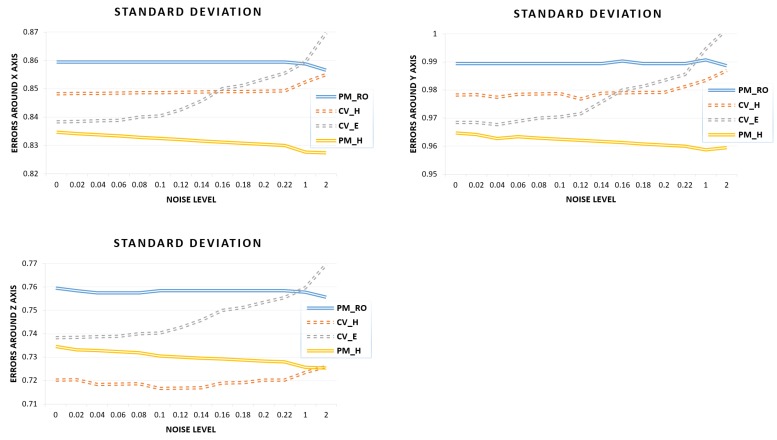
Error analysis of rotations around the *x*, *y*, and *z* axes for varying noises.

This experiment examines the stability of the all estimations with the real dataset. We chose n=50 random point correspondences as noise, nearing each of the six numbers: $\sigma_{1}=0.06$, $\sigma_{2}=0.1,\;\sigma_{3}=0.16,\;\sigma_{4}=0.2,\;\sigma_{5}=1,\;\mathrm{and}\;\sigma_{6}=2$, and computed the rotations around the *x*, *y*, and *z* axes. The experimental results, as illustrated in Figure 6, give the standard deviation of the rotation errors around the *x*, *y*, and *z* axes. From Figure 6, we observe that the non-linear approaches (PM_H and PM_RO) were more stable with increasing noises, as compared to the linear approaches (CV_H and CV_E). We see that the linear approaches were critical and showed accuracy degradation with increasing noise. The experimental results strongly support the fact that the linear approaches are very sensitive to noise. The computation of CV_E became unstable when reaching the middle of the noise level range. We also observed that the errors from the computation of CV_H were stable at the low noise levels but, as the noise level increased, the stability decreased.

### 4.2. Performance Analysis for Estimations with a Simulated Dataset

We checked the accuracy of the estimated motion parameters for simulated datasets in two different test cases. We checked the accuracy of the estimated rotation parameters by comparing them with true (known) rotation parameters. For a true reference value, we created synthetic corresponding points between the template and the next consecutive frames of the 3D object. Using the created corresponding points, a precise 3D motion was estimated for each frame. In the first case, the 3D object in the simulated sequences was rotated along one of the *x*, *y*, or *z* axes separately, and translated along an *x*, *y*, or *z* axis. In the second case, the 3D object was rotated by a combination of rotation degrees around the *x*, *y*, and *z* axes, and translated by 15 units along one of the *x*, *y*, or *z* axes.

**Figure 7:**
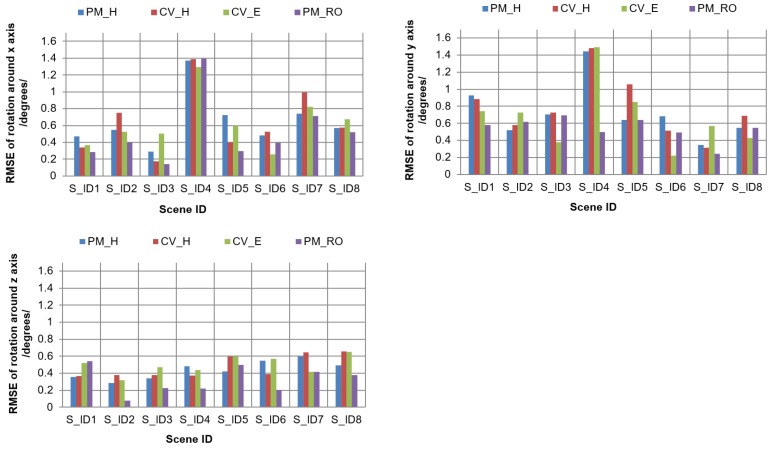
Comparisons of error analyses for rotations around the *x*, *y*, and *z* axes for simulated scenes.

For both cases, we checked the accuracy of the estimated rotation parameters through motion estimations. To check the performance of the four estimations, we compared the estimated rotation parameters with the known rotation parameters. We analyzed the mean, Max, and Min errors in the results of the estimations and summarize them in Table 6. We also computed the RMSEs of the estimated rotation results by comparing them against the true rotation parameters for each of the four estimations. The dataset summaries of RMSEs for rotations around the *x*, *y*, and *z* axes are given in Table 7, Table 8 and Table 9, respectively. A graphical representation of the comparisons is shown in Figure 7.

As we see in Table 6, Table 7, Table 8 and Table 9, the four estimations in photogrammetry and computer vision produced small errors in both test cases of the simulated datasets. The success rates of the estimations stayed within the range for large motions in the moving 3D object; this means that large perspective changes did not affect estimation accuracy. Moreover, this implies that all four estimations worked successfully when favorable corresponding points were provided. Specifically, the motion parameters obtained by PM_RO were the most accurate, compared to the other three approaches. It is confirmed, again, that PM_RO outperformed the other three approaches for datasets of 3D objects.

Generally, relative orientation-based approaches are formulated in non-linear problems, requiring an initial guess for each unknown parameter; however, these approaches are more robust to unique solutions for motion parameters with noisy point correspondences. Moreover, these approaches do not require additional computation to choose a correct solution for the motion parameters and estimate the rotation and translation parameters directly, compared with the linear approaches. Combining the two cases of test datasets, we observed that the PM_H approach produced more accurate results for planar objects in real-image sequences, and the PM_RO approach produced more accurate results for 3D objects in real and simulated sequences. On the other hand, the results with a real dataset are very interesting. Regardless of linear or non-linear approaches, homography-based methods outperformed other (essential matrix or relative orientation-based) methods under noisy situations. In particular, we observed that the homography-based non-linear approach worked better than the relative orientation-based non-linear approach. In other words, the homography-based non-linear approach supports the motivation for linking the techniques developed in photogrammetry and computer vision.

### 4.3. Qualification of Fast Processing

Once we define the template region in the initial frame, real-time processing is started with all computational steps. We extracted feature points by CPU-based or GPU-based parallel processing with a GeForce GTX 550 Ti graphics card.

To assess real-time performance, we measured the processing time for SIFT feature extraction with different numbers of extracted feature points. The speed of feature extraction was almost independent of the number of feature points, due to parallel processing. The processing time in SIFT feature extraction speeds up with a large number of feature points, by 0.2 s. Moreover, processing time can speed up with more powerful CPUs and graphics cards.

We also measured the processing time of four motion estimations, by including RANSAC-based elimination of outliers for different numbers of correspondences. For this, the comparison results are summarized in Table 10.

As we can see in Table 10, the processing times of all four methods were very fast with large numbers of feature correspondences. Generally, the processing speed of the PM_H estimation method is slower than the other three methods, due to its non-linear estimation. The processing speeds of the PM_RO and CV_H functions are the fastest. The total processing time could be defined as the sum of processing times for feature extraction and estimation of motion parameters.

## 5. Conclusions

In this study, we investigated and compared pose estimations in computer vision and photogrammetry. All estimations were implemented with common datasets and processing steps, which are suitable to use with a single camera. The results from the comparisons demonstrated the main differences in, and computational behavior of, the approaches developed by the computer vision and photogrammetry communities. In order to check the performance of these methods, we estimated the 3D motion of moving planar and 3D objects, at the experimental level, by using corresponding points between a template region and subsequent frames. Outlier corresponding points were eliminated by different RANSAC-based methods, which were adapted for each estimation. The experimental results were evaluated for the measured corresponding points, which were measured manually for both synthetic and real images. The results of the estimations in both fields were accurate, even with high variations of translation and rotation changes, with decent point correspondences. For noisy situations, the methods based on homography produced smaller errors. Comparisons of both fields highlight the robust performance of the estimations and 3D applications in each field. The processing speed was close to real-time. Due to the motion of the moving objects, the estimations diverged or converged to the correct solution. In further research, we plan to determine which method is more suitable in estimating the motions in a moving object.

## Figures and Tables

**Table 1:** Datasets used in the experiments.

Dataset for Real Scenes									
	**Dataset for 3D Objects**					**Dataset for Planar Objects**			
**Dataset ID**	F_ID21	F_ID22	F_ID23	F_ID24	F_ID25	F_ID31	F_ID32	F_ID33	F_ID34
**Template**	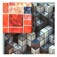	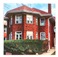	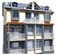	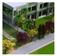	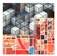	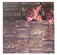	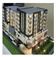	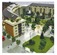	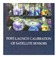
**Feature points**	115	104	78	151	159	62	86	189	126
**Size**	150×120								
**Dataset for Simulated Scenes**									
**Dataset ID**		S_ID1	S_ID2	S_ID3	S_ID4	S_ID5	S_ID6	S_ID7	S_ID8
**3D object**		polygon	cube	pyramid	polygon	cube	polygon	cube	pyramid
**Edge length**		15	15	15	13	13	10	10	10
**Feature points**		15	25	25	13	25	21	15	16

**Table 2:** Comparison of error analysis for rotation around *x*, *y*, and *z* axes for real scenes.

	Comparison of 3D Objects /Degrees/				Comparison of Planar Objects /Degrees/			
	PM_H	CV_H	CV_E	PM_RO	PM_H	CV_H	CV_E	PM_RO
**Max**	1.867	1.993	1.997	1.863	1.828	1.837	1.801	1.983
**Min**	0.00045	0.001	0.005	0.0004	0.0001	0.00014	0.013	0.0009
**Mean**	0.538	0.564	1.096	0.527	0.312	0.391	0.677	0.408

**Table 3:** Comparison of error analysis for rotation around *x* axis for real scenes.

RMSEs of the Estimated Rotation around the *x* axis ($\omega =1^{\circ }∼20^{\circ }$ Degrees)					
Dataset		PM_H	CV_H	CV_E	PM_RO
**F_ID21**	$\omega =5^{\circ },B_{y}=-5$ cm	0.103	0.146	0.560	0.112
**F_ID22**	$\phi =6^{\circ },B_{y}=20$ cm	0.465	0.488	0.551	0.530
**F_ID23**	$\omega =10^{\circ },B_{x}=-10$ cm	0.144	0.155	0.878	0.168
**F_ID24**	$\phi =13^{\circ },B_{x}=15$ cm	0.228	0.636	0.896	0.215
**F_ID25**	$\omega =4^{\circ },B_{z}=8$ cm	0.411	0.484	0.778	0.522
**F_ID31**	$\phi =15^{\circ },B_{y}=-13$ cm	0.065	0.068	0.647	0.127
**F_ID32**	$\kappa =20^{\circ },B_{x}=-10$ cm	0.100	0.108	0.233	0.143
**F_ID33**	$\phi =15^{\circ },B_{z}=-15$ cm	1.012	1.013	1.167	1.003
**F_ID34**	$\kappa =20^{\circ },B_{x}=10$ cm	0.165	0.233	0.991	0.415

**Table 4:** Comparison of error analysis for rotation around the *y* axis for real scenes.

RMSEs of the Estimated Rotation around the *y* axis ($\phi =1^{\circ }∼20^{\circ }$ Degrees)					
Dataset		PM_H	CV_H	CV_E	PM_RO
**F_ID21**	$\phi =5^{\circ },B_{z}=-8$ cm	0.458	0.488	0.477	0.627
**F_ID22**	$\omega =6^{\circ },B_{y}=9$ cm	0.429	0.106	0.302	0.101
**F_ID23**	$\omega =10^{\circ },B_{x}=-13$ cm	0.195	0.161	1.001	0.128
**F_ID24**	$\phi =13^{\circ },B_{x}=18$ cm	0.335	0.401	0.601	0.358
**F_ID25**	$\omega =15^{\circ },B_{z}=5$ cm	0.381	0.381	0.847	0.323
**F_ID31**	$\kappa =15^{\circ },B_{z}=-7$ cm	0.224	0.116	0.947	0.716
**F_ID32**	$\omega =20^{\circ },B_{y}=-6$ cm	0.681	0.790	0.954	0.552
**F_ID33**	$\kappa =15^{\circ },B_{x}=15$ cm	0.163	0.287	1.048	0.298
**F_ID34**	$\phi =20^{\circ },B_{z}=-20$ cm	1.323	1.377	1.62	1.535

**Table 5:** Comparison of error analysis for rotation around the *z* axis for real scenes.

RMSEs of the Estimated Rotation around the *z* axis ($\kappa =1^{\circ }∼90^{\circ }$ Degrees)					
Dataset		PM_H	CV_H	CV_E	PM_RO
**F_ID21**	$\omega =5^{\circ },B_{z}=6$ cm	0.477	0.468	0.758	0.219
**F_ID22**	$\omega =6^{\circ },B_{y}=-4$ cm	0.419	0.475	0.537	0.532
**F_ID23**	$\kappa =10^{\circ },B_{x}=16$ cm	0.195	0.161	0.201	0.228
**F_ID24**	$\omega =13^{\circ },B_{z}=-7$ cm	0.535	0.521	0.6002	0.458
**F_ID25**	$\kappa =15^{\circ },B_{x}=-17$ cm	0.381	0.401	0.684	0.423
**F_ID31**	$\kappa =15^{\circ },B_{y}=-4$ cm	0.0001	0.0001	0.198	0.0001
**F_ID32**	$\phi =20^{\circ },B_{x}=-10$ cm	0.002	0.0006	0.046	0.103
**F_ID33**	$\kappa =10^{\circ },B_{z}=5$ cm	0.004	0.005	0.085	0.003
**F_ID34**	$\phi =20^{\circ },B_{z}=-10$ cm	0.472	0.482	0.615	0.495

**Table 6:** Comparison of error analysis for rotation around the *x*, *y*, and *z* axes for the simulated dataset.

Error Comparison for 3D Objects /Degrees/				
	PM_H	CV_H	CV_E	PM_RO
**Max**	1.547	1.515	1.631	1.489
**Min**	0.015	0.009	0.013	0.003
**Mean**	0.549	0.498	0.762	0.461

**Table 7:** Comparison of error analysis for rotation around the *x* axis for the simulated dataset.

RMSEs of the Estimated Rotations around the *x* axis ($\omega =1^{\circ }∼30^{\circ }$ Degrees)					
Dataset		PM_H	CV_H	CV_E	PM_RO
**S_ID1**	$\omega =5^{\circ },B_{x}=-13$	0.471	0.338	0.365	0.282
**S_ID2**	$\omega =10^{\circ },B_{y}=8$	0.546	0.750	0.528	0.402
**S_ID3**	$\omega =15^{\circ },B_{y}=-5$	0.290	0.173	0.506	0.141
**S_ID4**	$\omega =25^{\circ },B_{x}=10$	1.369	1.385	1.296	1.390
**S_ID5**	$\phi =14^{\circ },B_{z}=-3$	0.724	0.397	0.598	0.296
**S_ID6**	$\phi =15^{\circ },B_{z}=-5$	0.484	0.527	1.256	0.396
**S_ID7**	$\kappa =25^{\circ },B_{z}=7$	0.740	0.990	0.823	0.711
**S_ID8**	$\phi =20^{\circ },B_{z}=12$	0.569	0.575	0.675	0.52

**Table 8:** Comparison of error analysis for rotation around the *y* axis for the simulated dataset.

RMSEs of the Estimated Rotations around the *y* axis ($\phi =1^{\circ }∼30^{\circ }$ Degrees)					
Dataset		PM_H	CV_H	CV_E	PM_RO
**S_ID1**	$\phi =8^{\circ },B_{x}=-15$	0.929	0.881	0.743	0.581
**S_ID2**	$\phi =15^{\circ },B_{y}=-3$	0.517	0.577	0.725	0.616
**S_ID3**	$\phi =22^{\circ },B_{y}=3$	0.702	0.723	0.377	0.692
**S_ID4**	$\phi =28^{\circ },B_{x}=8$	1.442	1.481	1.492	0.497
**S_ID5**	$\omega =25^{\circ },B_{z}=-5$	0.639	1.059	0.851	0.637
**S_ID6**	$\kappa =13^{\circ },B_{z}=-5$	0.681	0.513	0.221	0.493
**S_ID7**	$\kappa =18^{\circ },B_{z}=10$	0.344	0.313	0.568	0.245
**S_ID8**	$\kappa =15^{\circ },B_{z}=15$	0.548	0.687	0.425	0.549

**Table 9:** Comparison of error analysis for rotation around the *z* axis for the simulated dataset.

RMSEs of the Estimated Rotations around the *z* axis ($\kappa =1^{\circ }∼90^{\circ }$ Degrees)					
Dataset		PM_H	CV_H	CV_E	PM_RO
**S_ID1**	$\kappa =5^{\circ },B_{x}=-12$	0.353	0.369	0.522	0.542
**S_ID2**	$\kappa =6^{\circ },B_{y}=-2$	0.283	0.375	0.316	0.077
**S_ID3**	$\kappa =10^{\circ },B_{y}=5$	0.341	0.377	0.468	0.226
**S_ID4**	$\kappa =13^{\circ },B_{x}=13$	0.484	0.374	0.439	0.221
**S_ID5**	$\kappa =20^{\circ },B_{z}=-5$	0.424	0.597	0.598	0.496
**S_ID6**	$\phi =15^{\circ },B_{z}=-13$	0.549	0.387	0.571	0.205
**S_ID7**	$\phi =20^{\circ },B_{z}=6$	0.594	0.643	0.418	0.415
**S_ID8**	$\phi =15^{\circ },B_{z}=-15$	0.495	0.655	0.651	0.380

**Table 10:** Comparison of processing times for 3D motion estimation.

Processing Time /s/				
Feature Number	PM_H	CV_H	CV_E	PM_RO
227	0.002	0.0001	0.001	0.001
190	0.004	0.001	0.002	0.001
172	0.003	0.001	0.002	0.00001
156	0.003	0.001	0.002	0.001
110	0.004	0.0001	0.002	0.001
89	0.003	0.001	0.001	0.00001
46	0.003	0.001	0.001	0.00001
22	0.004	0.0001	0.0001	0.00001
9	0.019	0.0001	0.0001	0.00001
